# Urological cancer organoids, patients' avatars for precision medicine: past, present and future

**DOI:** 10.1186/s13578-022-00866-8

**Published:** 2022-08-19

**Authors:** Haotian Chen, Wentao Zhang, Niraj Maskey, Fuhan Yang, Zongtai Zheng, Cheng Li, Ruiliang Wang, Pengfei Wu, Shiyu Mao, Junfeng Zhang, Yang Yan, Wei Li, Xudong Yao

**Affiliations:** 1 Department of Urology, Shanghai Tenth People’s Hospital, School of Medicine, Tongji University, Shanghai, 200072 China; 2 Urologic Cancer Institute, School of Medicine, Tongji University, Shanghai, China; 3 Department of Urology, Guangdong Second Provincial General Hospital, Guangzhou, 510317 Guangdong China

**Keywords:** Organoids, Urological cancer, Organoid applications, Organoid challenges, Personalized medicine

## Abstract

Urological cancers are common malignant cancers worldwide, with annually increasing morbidity and mortality rates. For decades, two-dimensional cell cultures and animal models have been widely used to study the development and underlying molecular mechanisms of urological cancers. However, they either fail to reflect cancer heterogeneity or are time-consuming and labour-intensive. The recent emergence of a three-dimensional culture model called organoid has the potential to overcome the shortcomings of traditional models. For example, organoids can recapitulate the histopathological and molecular diversity of original cancer and reflect the interaction between cancer and surrounding cells or stroma by simulating tumour microenvironments. Emerging evidence suggests that urine-derived organoids can be generated, which could be a novel non-invasive liquid biopsy method that provides new ideas for clinical precision therapy. However, the current research on organoids has encountered some bottlenecks, such as the lack of a standard culture process, the need to optimize the culture medium and the inability to completely simulate the immune system in vivo. Nonetheless, cell co-culture and organoid-on-a-chip have significant potential to solve these problems. In this review, the latest applications of organoids in drug screening, cancer origin investigation and combined single-cell sequencing are illustrated. Furthermore, the development and application of organoids in urological cancers and their challenges are summarised.

## Background

Urological cancers include kidney, urothelial (including bladder, ureter and urethra), prostate, testicular and penile cancers. The morbidity and mortality of urological cancers are increasing annually. In 2021, 424,210 new cases of urological cancers and 66,970 cases of urological cancer-related deaths were predicted in the United States. Prostate cancer is one of the most common cancers and the third leading cause of death in men, joining renal and bladder cancers in the top 10 most common malignancies [1]. The China Cancer Centre estimates 92,000 urological cancer-related deaths in 2015, with prostate cancer being the third most common cause of mortality among urban residents [2].

With advances in sequencing technologies and the study of the underlying molecular mechanisms of cancer, it has been elucidated that different cancers or subtypes have distinct genetic alterations. For example, the Luminal Papillary type in muscle-invasive bladder cancer mainly expresses FGFR3 and KDM6A mutations whereas that in basal/squamous type are TP53 and RB1 mutations [3]. Some patients have drug resistance and high mortality due to rare gene mutations or intratumoral heterogeneity. Organoids, as a novel 3D model, maintain the heterogeneity of parental cancer and simulate the real environment in vivo, which highlights their potential for identifying therapeutic targets and verifying drug response [4]. The main sources of organoids are cell lines, adult stem cells and iPSCs; however, emerging evidence has shown that kidney organoids can also be generated from urine [5]. This breakthrough enables non-invasive access to patient-derived organoids (PDOs) and aids to develop novel therapeutic approaches. Tumour microenvironments (TME) have profound effects on cancer proliferation, differentiation, migration and invasion [6], indicating that no cell in our body is an island. Co-culturing components of the TME with organoids to study the connection between cancer and the surrounding environment is a commonly used research method [7]. Nevertheless, there is an emerging need for innovative engineering approaches for the production, control and analysis of organoids and their microenvironment. Furthermore, the organoid-on-a-chip technology produced by the combination of organoids and microfluidics could be an effective means to solve these problems [8–10]. With the development and popularization of single-cell sequencing technology, researchers can analyse the genetic information of organoids at single-cell resolution, which could aid in further understanding the application of organoids [11]. In this review, the development of urological cancer organoids and their advantages over cell lines and xenotransplantation are described, and the latest applications of urological cancer organoids are presented. Furthermore, emerging opportunities and future obstacles to the development and application of organoids are discussed.

## Evolution of culture methods

### Cell line cultures and xenotransplantation

Traditional culturing involves mainly cell line cultures, which describe genetic variation at the cellular level. However, with advancements in technology, the limitations in cell line cultures are more evident. For example, cell lines mimic the extracellular matrix but not the cell-to-cell interactions that occur in a 3D environment [12, 13]. These interactions are an important part of the TME and are associated with cancer occurrence, development and immune evasion. For many years, cell line cultures were unable to replicate the genetic representation, morphology, polarity and cytoskeletal components of cancer. Some of the major features of cancer such as hypoxia, nutrient gradients and drug penetration were also not reflected. Additionally, long-term culture could result in altered genetic information [14, 15]. This hinders the study of cancer heterogeneity and affects the development of individualized treatments.

Animal models are another important method to study cancers [16]. In the last century, Druckreyet et al. demonstrated that a subcutaneous injection of 4-hydroxybutylnitrosamine can replicate natural carcinogenesis in rodents to induce bladder cancer. The patient-derived xenografts (PDXs) model, which has become popular recently, involves the subcutaneous or orthotopic implantation of human cancer cell lines or freshly resected patient cancer specimens into immunodeficient nude mice. The subcutaneous PDXs allow researchers to monitor tumour growth and assess anti-cancer drug responses. The orthotopic PDXs can partially simulate the local microenvironment and mimic cell-to-cell interactions; however, monitoring cancer growth is difficult, unless using expensive imaging equipment (e.g.: computed tomography and magnetic resonance imaging). PDXs have been used to evaluate the efficacy of various therapeutic approaches such as intravesical therapy for bladder cancer [14, 16]. However, PDXs still have some drawbacks such as unsuitability for gene editing; being time-consuming and expensive; difficulty in performing high-throughput drug screening; significantly different TME of subcutaneous models than that of naturally occurring human cancers; low transplant success rates; and different genetic backgrounds. Therefore, as a human cancer model, PDXs have considerable limitations.

### Organoid culture system

‘Organoid’ is a type of culture system wherein cells from specific organs form small clumps. They can self-organize through cell sorting and limited lineage synthesis, replicate the histological and genetic features of these organs and reveal their new functions [17]. In 2009, Sato et al. [17, 18] found that a single intestinal stem cell can generate a physiological epithelial structure that continuously expands and reorganizes itself. The intestinal stem cell had G protein-coupled receptor 5 (Lgr5) positive and leucine-rich repeats, which closely resembled normal intestinal tissue, hence, the name ‘organoid’. They embedded the stem cells in Matrigel containing R-spondin1, Noggin and epidermal growth factor (EGF). R-spondin1 activates WNT signalling, which is required for crypt proliferation, by binding to Lgr5; Noggin inhibits bone morphogenetic protein (BMP) signalling and increases crypt number; EGF is a growth factor necessary for crypt growth. Since then, these three factors have been widely used in organoid cultures. Liu et al. [19] and Chapman et al. [20] subsequently demonstrated that the combination of the Rho-related protein kinase (Rock) inhibitor Y-27632 and a fibroblast feeder layer supported the indefinite growth of various primary human epithelial cells. Thus, organoids derived from normal cells or cancer cells could proliferate immortally in vitro without the transfection of exogenous viral or cellular genes. These studies have laid a solid foundation for the establishment of a urological cancer organoid medium. Jarno et al. [21] developed a fully defined serum-free medium for prostate cancer organoids that contain pluripotent stem cells. These organoids can be cultured for long periods and contain androgen receptor signalling. Furthermore, Jasper et al. [22] developed a stable human bladder cancer organoid medium based on previous reports on primary bladder urothelium cancer and organoid media. They observed that fibroblast growth factor 7 (FGF7) and FGF10 stimulated the proliferation of human bladder cancer organoids and that the medium had an approximately 50% success rate in establishing human bladder cancer organoids. The organoids can be sub-cultured weekly and propagated for long periods (> 1 year). Based on studies of prostate cancer and bladder cancer organoids, Annika et al. [23] established clear cell renal cell carcinoma (ccRCC) organoids that had better expansion capacity, a higher success rate and more epithelial polarization compared with previous organoids [24]. In addition to the commonly used medium components, they also added amphotericin B and heparin (Table 1).

**Table 1:** Composition of the culture medium for urological cancer organoids

Types of organoids	Culture medium
Generic ingredients	DMEM/F12
	EGF
	Y-27632(ROCK inhibitor)
	A83-01
Prostate cancer	Prostaglandin E2
	DHT
	B27
	N-acetylcysteine
	Noggin
	R-spondin1
	FGF10
	FGF2
	Nicotinamide
	SB202190
Bladder cancer	FGF7
	FGF10
	N-acetylcysteine
	Nicotinamide
	N-21
	Heregulin
	CHIR99021
Renal cancer	Heparin
	Amphotericin B
	bFGF
	B27
	N-acetylcysteine

*EGF* epidermal growth factor, *ROCK* Rho-related protein kinase, *DHT* dihydrotestosterone, *FGF* fibroblast growth factor

Recently, organoids have been increasingly used as a model to study cancer genetics, including those of the brain [25], thyroid [26], breast [27], pancreas [28], liver [29], lung [30] and colon [31]. Moreover, the use of organoids in urological cancers is expanding especially in bladder, prostate and renal cancers [32–34]. Organoids can make up for the limitations of cell line cultures and xenotransplantation. It can reconstruct the TME by simulating cell-to-cell and cell-extracellular matrix interactions in vivo to further study cancer physiology and pathogenesis. Organoids are also a convenient model for high-throughput drug screening and gene sequencing and can be combined with experimental methods, including immunohistochemistry, immunofluorescence and single-cell sequencing [35]. The advantage of an organoid lies in its human genetic background, which effectively reduces the deviation of experimental results caused by genetic differences between species compared with cell lines and animal models (Table 2).

**Table 2:** Comparison of cell lines, PDXs, and organoid

	Cell line	PDX	Organoid
Advantages	1. Facilitate genetic manipulation 2. High-throughput drug screening 3. Easy cultivate, high success rate 4. Cheap and time saving	1. Retains the phenotypic and genetic heterogeneity 2. Simulate the local TME and cancer-host interaction 3. Easy to observe drug response	1. Retains the genetic characteristics and phenotypic 2. Simulate partial TME 3. High-throughput drug screening 4. Facilitate gene manipulation and functional studies
Limitations	1. Lack of genetic heterogeneity 2. Unable to reflect the phenotype 3. Unable to simulate the TME 4. Long-term culture is prone to genetic drift	1. Difficulty in high-throughput screening 2. Difficulty in gene editing and functional studies 3. Different genetic backgrounds 4. Time- consuming, low success rate and expensive	1. Lack of blood vessels and a complete immune system 2. The components in the culture medium may have potential effects on cancer drug response 3. Low success rate of long-term cultivation

### Development of organoids in urological cancers

**Fig.1:**
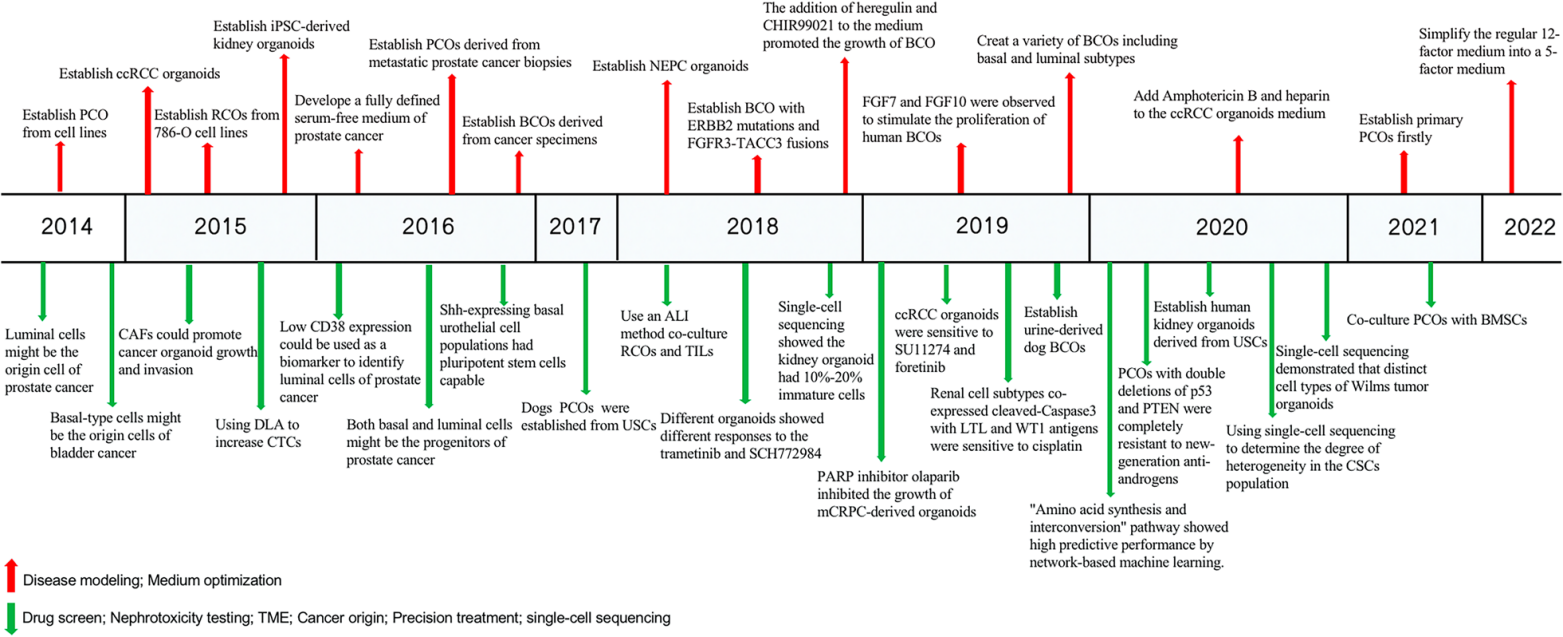
The development of urological cancer organoids and media in the form of a year chart. Red arrows represent the establishment of urological cancer organoids and optimization of the media; Green arrows represent the applications of urological cancer organoid

The development of organoids in urological cancers is mainly concentrated in prostate, bladder and renal cancers. Currently, the main sources for establishing organoids are cell lines and adult stem cells from cancer biopsy or surgery. The iPSC-derived kidney organoids can aid in understanding the development of the kidney and the transformation mechanism of cancer. Based on the development of organoids from urine, urine stem cells (USCs) derived organoids are yet to be determined if they can completely retain the characteristics of original cancer and can be stably cultured. This research focus has the potential to greatly promote the development of precision medicine and individualized therapy. The establishment of organoids is inseparable from suitable media and different cancer organoids require special additives. We described the general process of the development of urological cancer organoids below. (Fig. 1) (Table 3).

**Table 3:** Research applications of urological cancer organoids

Year	Author	Application	Model	Mainly findings	Refs
2014	Gao et al.	Disease modeling	PCO	Human prostate cancer organoids have been established for the first time and these organoids can reproduce the characteristic copy number and mutation profiles commonly found in prostate cancer	[40]
2016	Drost et al.	Disease modeling	PCO	They first cultured organoids derived from human metastatic prostate cancer biopsies and describe a specific culture protocol	[21]
2018	Park et al.	Disease modeling	NEPC organoid	Generation of NEPC organoids from benign prostate epithelial cells	[41]
2021	Servant et al.	Disease modeling	PCO	Furthermore, developed a biobank consisting of PCOs derived from 81 patients with primary and metastatic prostate cancer. A significant correlation was observed between the percentage of tumour cancers in the parental sample and organoid growth, implying that tumour purity could be a predictive factor for organoid growth	[42]
2016	Justin et al.	Disease modeling	BCO	They established 4 organoid lines derived from cancer specimens and found that organoids exhibited mutations that were highly concordant with original cancer samples	[51]
2018	Lee et al.	Disease modeling	BCO	Bladder cancer organoids harboring ERBB2 mutations and FGFR3-TACC3 fusions were established and their mutational, molecular and histopathological features were highly consistent with original cancers	[52]
2019	Mullenders et al.	Disease modeling	BCO	They created a variety of BCOs including basal and luminal subtypes. Moreover, all mouse and most human BCOs contained cells that stained positive for keratin 5 and CD44, which are two potential cancer stem cell markers	[22]
2015	Takasato et al.	Disease modeling	Kidney organoid	They established iPSC-derived kidney organoids	[67]
2015	Pan et al.	Disease modeling	RCO	They established renal cancer organoids from 786-O cell lines. These organoids expressed ccRCC characteristic gene CXCR4 and selective adhesion molecules, angiogenic factors and osteolytic factors related to bone metastasis	[68]
2015	Batchelder et al.	Disease modeling	RCO	20 renal cancer organoids were established from 25 ccRCC samples	[32]
2016	Drost et al.	Medium optimization	PCO	A fully defined serum-free medium for prostate cancer organoid was developed in which organoids contain pluripotent progenitor cells; can be cultured long-term; and contain the androgen receptor signaling pathway	[21]
2022	Cheaito et al.	Medium optimization	PCO	They simplified the regular 12-factor medium into a 5-factor medium. The 5-factor medium was found to increase the number and size of prostate cancer organoids, but prolonged culture time. The addition of R-spondin1 can significantly shorten the culture time	[46]
2019	Mullenders et al.	Medium optimization	BCO	A stable human bladder cancer organoid culture medium was developed and FGF7 and FGF10 were observed to stimulate the proliferation of human bladder cancer organoids	[22]
2018	Yoshida et al.	Medium optimization	BCO	The addition of heregulin and CHIR99021 to the medium promoted the growth of bladder cancer organoids	[15]
2020	Fendler et al.	Medium optimization	RCO	Amphotericin B, and heparin are added to the culture medium of ccRCC organoids and are widely used	[23]
2020	Elbadawy et al.	Drug screening	PCO	They demonstrate that PTEN deletion enhances resistance to next-generation anti-androgens, and that prostate cancers with double deletions of p53 and PTEN will be completely resistant to new-generation anti-androgens	[72]
2019	Chakraborty et al.	Drug screening	PCO	It was found that co-deletion of BRCA2 and RB1 induces EMT and is associated with cancer aggressiveness and progression; the PARP inhibitor olaparib inhibits the growth of mCRPC-derived organoids and promotes cancer cell apoptosis	[74]
2018	Lee et al.	Drug screening	BCO	Different organoids showed different responses to the MEK inhibitor trametinib and the ERK inhibitor SCH772984. Muscle-invasive carcinomas and tumors that recur after treatment failure showed greater drug resistance	[52]
2020	Kong et al.	Drug screening	BCO	They found the "amino acid synthesis and interconversion" pathway showed high predictive performance by network-based machine learning	[75]
2019	Grassi et al.	Drug screening	RCO	They demonstrated that ccRCC organoids were sensitive to SU11274 and foretinib and had reduced pAKT and pERK gene expression. Furthermore, forratinib sustained apoptosis in ccRCC organoids	[33]
2019	Grassi et al.	Nephrotoxicity testing	Renal organoid	They found that renal cell subtypes in which cleaved-Caspase3 co-expressed with LTL and WT1 antigens were sensitive to cisplatin. But only the tubular cells were damaged	[33]
2015	Åkerfelt et al.	TME	PCO	Using a real-time live-cell measurement platform, they observed that CAFs promote cancer organoid growth and invasion. And found that FAK inhibitors Y11 and PF-573228 selectively disrupted cancer-CAFs interactions, inhibited caner growth and invasion, and had no apparent cytotoxicity [46]	[83]
2021	Dhimoleaet et al.	TME	PCO	Co-culturing prostate cancer organoids with BMSCs and found that IL-6 secreted by BMSCs induced hormone-independent growth of prostate cancer organoids by activating the JAK/STAT signaling pathway and the model was less sensitive to enzalutamide	[84]
2018	Neal et al.	TME	RCO	They used an ALI method propagated PDOs from 100 human ccRCC biopsies or syngeneic immunocompetent mice as cancer epithelia with native embedded immune cells. The association in vivo between native TILs and cancer cells is preserved. They demonstrated that PDOs accurately preserved the original cancer TCR profile by 10 × Chromium single-cell sequencing. Both human and mouse cancer organoid TILs functionally exhibit activation, expansion and cytotoxicity responses to PD-1/PD-L1 checkpoint blockade	[85]
2014	Chua et al.	Cancer origin	PCO	They established mouse prostate cancer organoids using a prostate cancer luminal cell line and showed through lineage tracing that luminal cells facilitate organoid formation and generate basal cells in culture, suggesting progenitor properties of luminal cells	[88]
2016	Liu et al.	Cancer origin	PCO	Low CD38 expression can be used as a biomarker to identify luminal cells of prostate cancer	[90]
2016	Park et al.	Cancer origin	PCO	Both basal and luminal cells are thought to be progenitors of prostate cancer. Both basal and luminal cells can respond to the same oncogenic lesions to initiate tumorigenesis, but with different tumor phenotypes	[91]
2014	Choi et al.	Cancer origin	BCO	Basal-type bladder cancer was found to be more aggressive, metastatic, and less survival than the luminal type, and was more sensitive to cisplatin-based chemotherapy	[92, 93]
2016	Ohishi et al.	Cancer origin	BCO	Single cells from Shh-expressing cell derived organoids were able to self-renew and generating new organoids in subsequent cultures, suggesting that Shh-expressing basal urothelial cell populations include pluripotent stem cells	[54]
2015	Mout et al.	Precision treatment	PCO	Pioneering use of DLA to increase CTCs in patients with metastatic prostate cancer. This method provides a rich source for culturing organoids, enabling liquid biopsies	[96]
2017	Usui et al.	Precision treatment	Dog PCO	For the first time, they established urine cancer stem cells derived PCOs from dogs with prostate cancer. They observed expression of the epithelial cell marker E-cadherin in the organoids by immunofluorescence staining	[99]
2019	Elbadawy et al.	Precision treatment	Dog BCO	They generated dog bladder cancer organoids using urine samples. They found that the expression levels of MMP28, CTSE, CNN3, TFPI2, COL17A1 and AGPAT4 were specifically upregulated in dog BCOs	[100]
2020	Sun et al.	Precision treatment	RCO	They used 10% kECM to establish human USCs derived kidney organoids. And they demonstrated that USCs-derived kidney organoids were similar to HKCs derived organoids in morphology, histology and specific gene expression	[103]
2018	Wu et al.	Single-cell sequencing	Kidney organoid	Researchers compared single-cell transcriptomics of 83,130 cells from 65 kidney organoids with fetal and adult kidney cells by single-cell RNA sequencing. The results showed the organoid-derived cell types were immature with 10%-20% of the cells being non-renal cells and mostly neurons	[114]
2020	Calandrini et al.	Single-cell sequencing	Kidney tumor organoids	They established the first organoid biobank for paediatric cancers which included 54 kidney tumor organoids and matched normal kidney organoids. Using single-cell RNA sequencing and high-resolution 3D imaging, they demonstrated that Wilms tumor-derived organoids are composed of multiple distinct cell types including epithelial, stromal and blastocyst-like cells	[115]
2020	Fendler et al.	Single-cell sequencing	RCO	They used single-cell sequencing to determine the degree of heterogeneity within the kidney CSCs population. This yielded three subpopulations, two of which exhibited high expression of markers known to be associated with stem cells and kidney development	[23]

*PCO* prostate cancer organoid, *BCO* bladder cancer organoid, *RCO* renal cell carcinoma organoid, *NEPC* neuroendocrine prostate cancer, *ccRCC* clear cell renal cell carcinoma, *mCRPC* metastatic castration-resistant prostate cancer, *CAFs* cancer-associated fibroblasts, *BMSCs* bone marrow stromal cells, *PDOs* Patient-Derived Organoids, *TCR* T cell receptor, *TILs* tumor-infiltrating lymphocytes, *DLA* diagnostic leukotomy, *CTCs* circulating tumor cells, *kECM* kidney-specific extracellular matrix, *USCs* urine stem cells, *HKCs* human kidney cells, *3D* three-dimensional, *CSCs* cancer stem cells

### Prostate cancer

Over the past century, only seven prostate cancer cell lines have been established, six from biopsy samples of patients with metastatic prostate cancer and one from circulating tumour cells (CTCs). Although cell lines are easy to culture, they fail to reflect common recurrent genetic lesions in prostate cancer such as TMPRSS2-ERG and CHD1 deletions, SPOP and FOXA1 mutations [36]. To fix this research gap, Schroe et al. [37] firstly reported the transplantation of prostate cancer into nude mice in 1976. Moreover, a continuous transplantable prostate cancer xenograft in nude mice, named PC82, has been established [38]. However, prostate cancer xenotransplantation has a low success rate ranging from 20 to 75% in subcutaneous models [39]. Nonetheless, the emergence of organoids is a promising solution.

**Fig.2:**
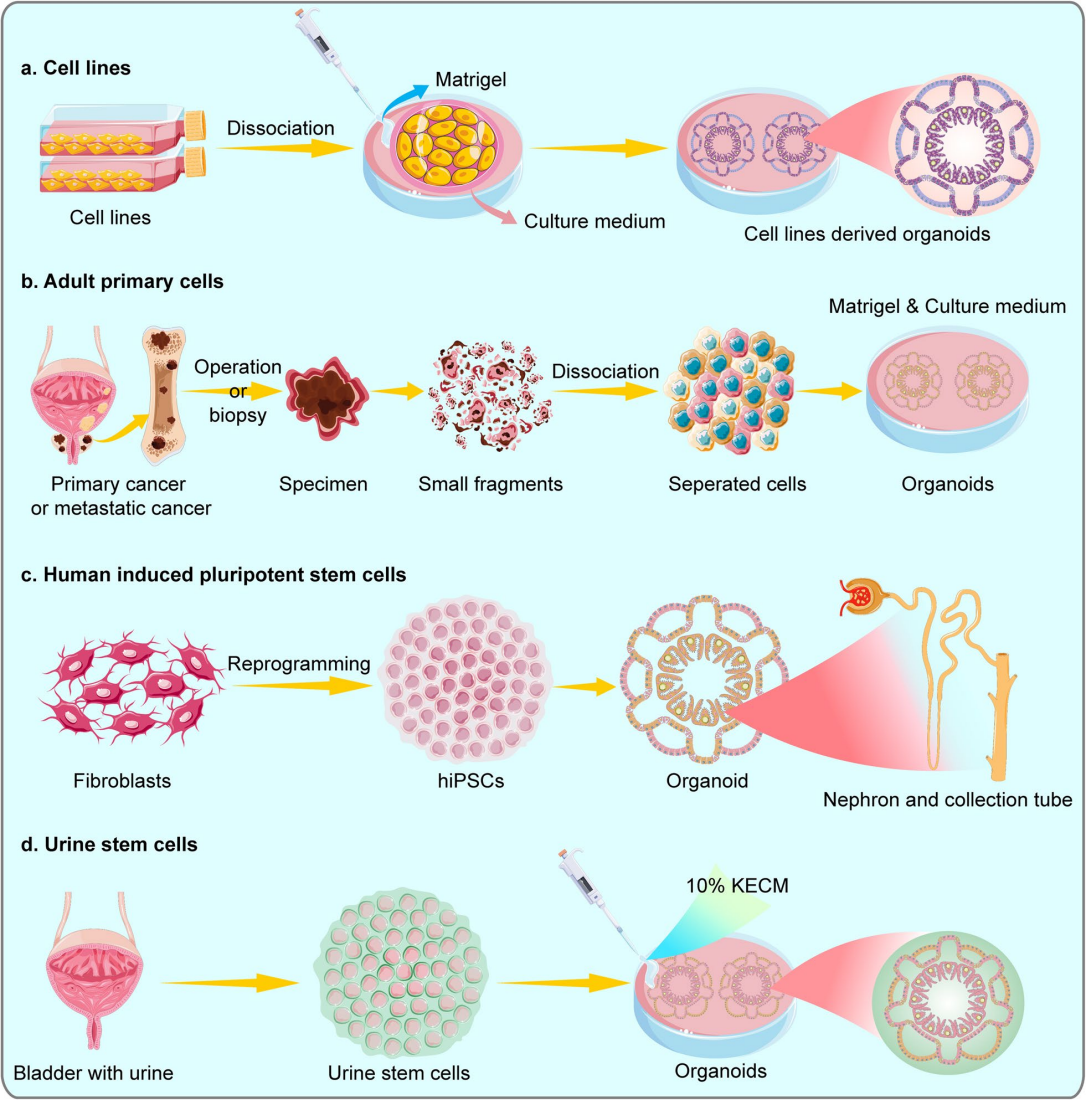
The development of organoids from different sources **a** Organoids from cell lines. Dissociated cancer cells can proliferate and survive long-term in Matrigel and culture medium. Organoids derived from prostate cancer cell lines can stably express common recurrent genetic lesions of prostate cancer. **b** Primary or metastatic tumour specimens obtained by operation or biopsy are cut into small fragments, dissociated into separated cells and then cultured into organoids. **c** Pluripotent stem cells can differentiate into any type of cell. Human-induced pluripotent stem cells (hiPSCs)-derived kidney organoids include distal and proximal tubules, early loops of Henle, glomeruli and collecting ducts. **d** Kidney organoids derived from human USCs have been established using a 10% kidney-specific extracellular matrix. These organoids express kidney products, such as AQP1 and EPO, and have partial kidney structure and function

In 2014, Gao et al. [40] first developed seven metastatic prostate cancer organoids (PCOs) using currently available cell lines and demonstrated that PCOs could reproduce characteristic copy number and mutation profiles commonly found in prostate cancer, including the loss of PTEN and CHD1, SPOP and FOXA1 mutations (Fig. 2). Additionally, PCOs are genetically highly consistent with original cancer and remain stable over time. In 2016, Jarno et al. [21] first cultured organoids derived from human metastatic prostate cancer biopsies and described a specific culture protocol. Their culture protocol allows the growth of both the luminal and basal prostatic epithelial lineages and the growth of advanced prostate cancers. Organoids established using this protocol can be used to study many different aspects of prostate cancer, including homeostasis, cancerogenesis and drug discovery. In 2018, Park et al. [41] generated neuroendocrine prostate cancer (NEPC) organoids from benign prostate epithelial cells via the lentiviral transfection of oncogenes, providing important insights into the drivers of prostate cancer progression. Furthermore, Servant et al. [42]. developed a biobank consisting of PCOs derived from 81 patients with primary and metastatic prostate cancer. They found radical prostatectomy (RP)‐derived organoids had higher efficiency and maintained longer term than transurethral resection of prostate (TURP)-derived organoids. A significant correlation was observed between the percentage of tumour cancers in the parental sample and organoid growth, implying that tumour purity could be a predictive factor for organoid growth. Therefore, larger sample cohorts are needed to identify predictive factors promoting these growth advantages.

There is no doubt that a suitable medium can improve the efficiency and success of an organoid culture. Matrigel serum-free medium supplemented with DHT and PGE2 constitutes an essential condition for PCO growth [43]. Some reports suggest that serum can be added to the medium, for example, the foetal bovine serum (FBS) can improve the success rate of the culture [44]. However, Gstrunthaler et al. [45] suggested that FBS could affect the consistency and results of experiments due to batch and source differences. Recently, Katia et al. [46] simplified the conventional 12-factor medium into a 5-factor medium and respectively added EGF, R-spondin1, FGF2, FGF10, PGE2 and SB202190 to the 5-factor medium. Compared with the 12-factor medium, the 5-factor medium increased the PCOs' number and size but prolonged the culture time. The addition of R-spondin1 can significantly shorten the culture time and increase the organoids number and size. These studies highlight the importance of the medium, underscoring that further research is needed to determine the optimal medium.

### Bladder cancer

Currently, few models can objectively mimic the biology of normal urothelium or bladder cancers [47–50]. Patient-derived bladder cancer organoids (BCOs) are expected to be a novel model that can elucidate the mechanism of bladder cancer development and drug response. Justin et al. [51] collected cancer specimens using cystoscopy and enzymatically digested them into single cells and cell clusters before culturing in organoid-promoting embedded cell culture conditions. Four organoid lines were established, and MSK-IMPACT was used to sequence cancer specimens and organoids. The organoids formed exhibited mutations that were highly concordant with original cancer samples. Of the four organoid lines sequenced, three have been successfully thawed and propagated further. In 2018, Lee et al. [52] established a BCO containing ERBB2 mutations and FGFR3-TACC3 fusion. They analysed 22 patient-derived muscle-invasive BCOs and demonstrated that mutational, molecular and histopathological features were highly consistent with original cancers. Further, the organoids not only retained significant tumour heterogeneity but also readily underwent clonal evolution in culture. Clonal evolution, which involves genetic changes in cancers over time, is a major contributor to cancer progression and drug resistance. On analysing phylogenetic trees, linear and branched patterns of organoids’ clonal evolution were observed that were similar to patterns described for urothelial cancers in vivo [53]. Based on previous studies, Mullenders et al. [22] optimized the culture medium to create a variety of BCOs including basal and luminal subtypes. BCOs were found to retain the characteristics of different bladder cancer subtypes by comparing biomarkers (such as Ck5, Ck20 and p63) in various subtypes of bladder cancer tissues and organoids in both mice and humans. Moreover, all mouse and most human BCOs contained cells that stained positive for keratin 5 and CD44, which are two potential cancer stem cell markers [54, 55]. Further analysis of BCOs can shed light on the actual stem cell in urothelial carcinoma.

Culturing BCOs requires placing cell suspensions from cell lines or cancer specimens in a medium containing growth factor and a cell matrix. Appropriate medium and cultural methods are crucial for the culture of BCOs. Takahiro et al. [15] found that the addition of heregulin and CHIR99021 to the culture medium promoted the growth of BCOs. Heregulin is a ligand for human epidermal growth factor receptor (HER) 3 and HER4; a tyrosine kinase receptor that transduces mitotic signalling. Meanwhile, CHIR99021 activates the Wnt pathway by inhibiting GSK3 and the subsequent phosphorylation and degradation of β-catenin. However, the growth-promoting abilities of heregulin and CHIR99021 are significantly distinct in different patient-derived BCOs, with varying success rates across tissues and subtypes. Papillary BCOs have a success rate of up to 80% while non-papillary BCOs have a success rate of less than 30% [56], which could be attributed to cancer heterogeneity.

### Renal cancer

Before 2006, only embryonic stem cells (ESCs) could be used to differentiate different cell types in vitro. However, research on ESCs is scarce owing to ethical reasons. In 2006, Takahashi et al. [57] obtained induced pluripotent stem cells (iPSCs) by reprogramming mouse fibroblasts. The pluripotency of iPSCs and ESCs are comparable, with both having the ability to differentiate into the three germ layers. In 2007, they used the same method to successfully construct human induced pluripotent stem cells (hiPSCs), which form the foundation for the cell source for building organoids. The induction of ESCs or iPSCs to establish renal organoids requires mimicking the process of kidney development under physiological conditions. Many signalling pathways play an important role in this process. For example, the Wnt signalling pathway plays a key role in balancing the expansion, differentiation and mesenchymal transition of kidney progenitor cells [58, 59]. FGF signalling is critical in maintaining the stemness of kidney progenitor cells [60] while BMP4 inhibits ectopic ureteral bud formation [61]. The mammalian kidney is derived from the intermediate mesoderm, which gives rise to key renal progenitor cell populations–the ureteral epithelium and metanephric mesenchyme. The ureteral epithelium and metanephric mesenchyme form collecting ducts and nephrons, respectively. Furthermore, several studies have reported the successful differentiation of hiPSCs into ureteral epithelium or metanephric mesenchyme in vitro [62–66]. Subsequently, Takasato et al. [67] identified a developmental mechanism of preferential induction between hiPSCs-induced collecting ducts and kidney mesenchymal progenitors by adding CHIR99021. Thus, hiPSCs-derived kidney organoids containing nephrons and collecting ducts were established. In these organoids, individual nephrons were segmented into distal and proximal tubules, early loops of Henle and glomeruli containing podocytes with elaborating foot processes and undergoing vascularization. When the transcription profiles of kidney organoids were compared to human foetal tissues, they showed the highest congruence with a first-trimester human kidney.

In 2015, Tianhong et al. [68] cultured the ccRCC bone metastases-derived 786-O cell lines in a hyaluronic acid hydrogel-based 3D culture system, wherein they proliferated spherically and had a long-term survival rate. Compared with 2D cell lines, 786-O-derived organoids expressed ccRCC characteristic gene CXCR4 and selective adhesion molecules, angiogenic factors and osteolytic factors related to bone metastasis [69–71] but exhibited a lower proliferation rate, indicating that the most organoids can better simulate the real situation of tumours in vivo. In the same year, Batchelder et al. [32] successfully established 20 cancer organoids from 25 ccRCC samples and 22 benign organoids from non-cancer samples using 3D scaffolds. Gene expression in these cells was maintained for up to 21 days. In 2020, Joon et al. [34] created an organoid culture method using samples from patients with advanced ccRCC based on previous protocols regarding renal tubular organoids. These organoids had similar histological characteristics and cell aggregation patterns to ccRCC. Furthermore, they well preserved the morphological features of ccRCC, including lipid-rich, clear cytoplasm and diverse colony formation. Additionally, the expression of biomarkers in organoids was investigated using immunofluorescence, RT-PCR and Western blot. The expression of classical cancer markers, such as CA9 and vimentin, was increased while the expression of keratin was decreased, which is consistent with the expression profile in ccRCC. Thus, these organoids are superior to cell line cultures in preserving the biomarker expression of original cancer. Therefore, as this ccRCC organoid model retains the morphological, histological and biological characteristics of the original cancer, it lays a foundation for precision therapy research.

## Application in urological cancers

### Drug screening and nephrotoxicity

**Fig.3:**
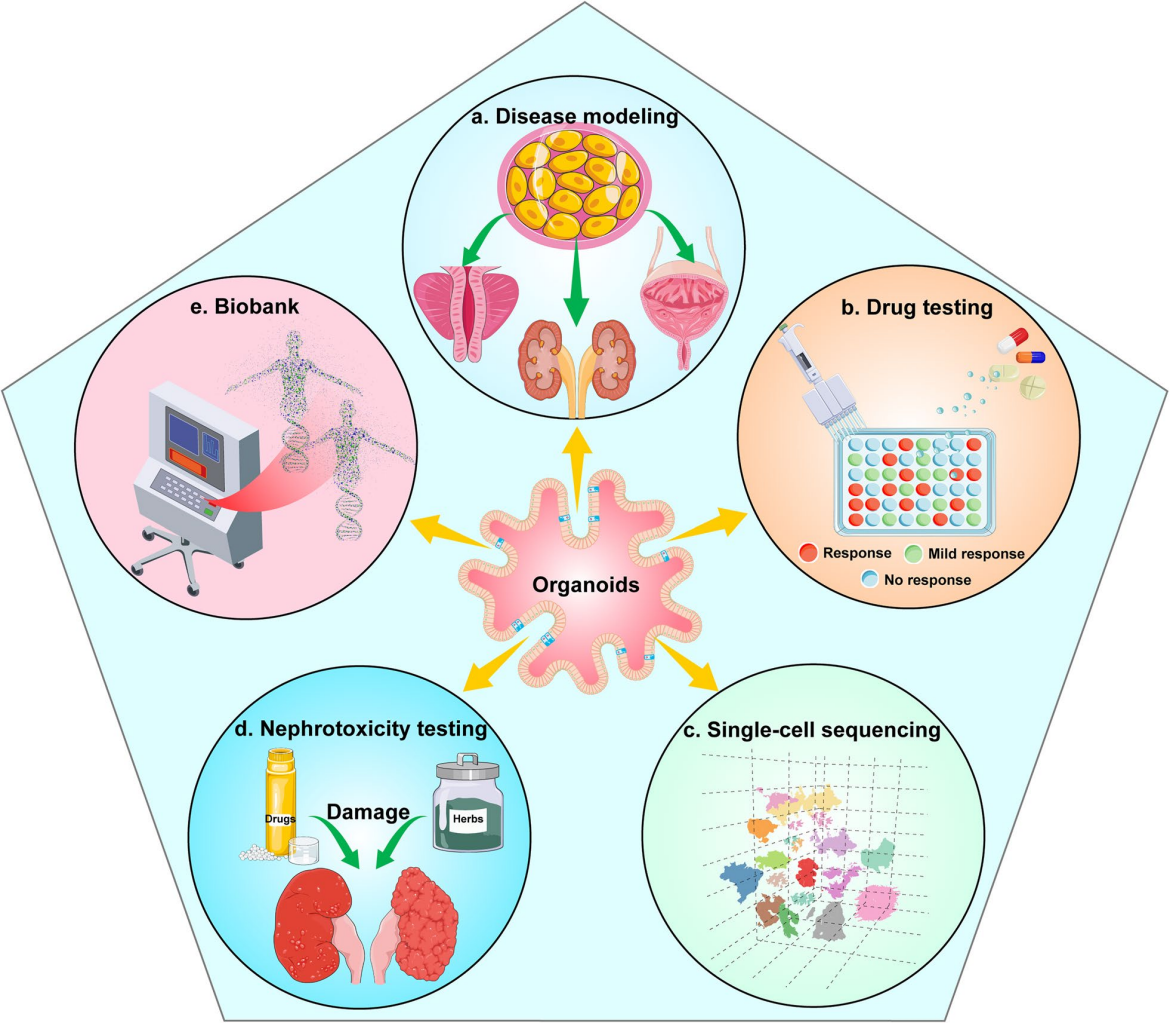
Diverse applications of urological cancer organoids **a** Tumour tissues or cell lines are used to generate patient-derived organoids (PDOs) and whole-genome sequencing is used to demonstrate that PDOs have the same genetic characteristics as primary tumours. Therefore, PDOs can be used to study cancer mechanisms and personalize medicine for precision treatment. **b** Urological cancer organoids can be used to study drug resistance and sensitivity to guide clinical treatment. **c** Single-cell sequencing technology studies the development and differentiation of cells by analysing the genetic material of each cell. Organoid technology is continuously improved by using single-cell sequencing to understand how similar organoids are to real organs. **d** The PDOs provide insight into the nephrotoxicity mechanism of drugs (such as cisplatin). **e** A large number of organoids were collected to build a rich biobank for drug screening and translational medicine

In castration-resistant prostate cancer (CRPC), a new generation of androgen deprivation therapy (ADT), such as abiraterone and enzalutamide, has been used in clinical treatment with great success. CRPC organoids with AR amplification, PIK3R1 mutation and PTEN deletion exhibit high sensitivity to enzalutamide [72, 73]. Nonetheless, some patients with CRPC eventually show insensitivity to various anti-androgen drugs, leading to ineffective treatments. Now, organoids can serve as a new platform to study the underlying mechanisms of cancer resistance (Fig. 3). Elbadawy et al. [72] studied the effect of anti-androgen drugs on mouse PCOs and observed that the loss of PTEN enhanced resistance to new generation anti-androgens. Furthermore, organoids with a double deletion of p53 and PTEN were completely resistant to the new generation of anti-androgen drugs. Thus, PCOs can provide new ideas for clinical drug screening and analysis of drug resistance. Recent sequencing studies have shown that DNA damage response (DDR)-related genetic alterations are more common in men with metastatic castration-resistant prostate cancer (mCRPC). BRCA2, a DDR and cancer susceptibility gene, is frequently deleted in mCRPC. Goutam et al. [74] found that patients with BRCA2-deficient prostate cancer often had a co-deletion of the cancer suppressor gene RB1 and the deletion of both genes induced epithelial-mesenchymal transition (EMT), which is associated with cancer aggressiveness and progression. Notably, the poly ADP ribose polymerase (PARP) inhibitor olaparib was found to inhibit the growth of human-derived mCRPC organoids and promote cancer cell apoptosis. This could be a potential novel treatment strategy for mCRPC.

Patient-derived BCOs represent a novel and superior model that better reflect the physiology of cancer growth and allow better identification and assessment of cancer development compared to cell culture. To explore the usefulness of organoids as a preclinical model for assessing drug response, Lee et al. [52] conducted a dose titration experiment exploring the effects of 40 drugs on nine organoids. The effects of trametinib and gemcitabine on BCOs were investigated, and BCOs before and after therapy were established. The organoids’ drug response was verified using orthotopic xenografts. Cancer histological changes and cell proliferation were significantly reduced after trametinib treatment whereas cleaved caspase-3 expression was significantly increased after gemcitabine treatment. This suggests that the two drugs can effectively inhibit the malignant proliferation of bladder cancer and promote cell apoptosis. Interestingly, different organoid lineages have significant similarities and differences among them as well as partial correlations to their mutational profiles. For example, some organoids with FGFR3 mutations showed significant responses to the MEK inhibitor trametinib and the ERK inhibitor SCH772984, whereas some organoids did not respond significantly to trametinib or SCH772984. None of the organoids showed responses to the three different FGFR inhibitors, which indicates a lack of potent stimulators or involves a more complex molecular mechanism. Additionally, they also used 26 compounds like trametinib and erlotinib for a drug response assay, measuring cell viability after six days of drug exposure using the CellTiter Glo assay. The muscle-invasive and recurrent bladder cancer-derived organoids were found to be insensitive to drug doses and exhibited strong drug resistance. Kong et al. [75] analysed the pharmacogenomic data of BCOs through network-based machine learning (ML) and found that the ‘amino acid synthesis and interconversion’ pathway showed high predictive performance across multiple ML algorithms. Therefore, it was selected as a biomarker of cisplatin response in bladder cancer. Furthermore, the reliability of this marker in cisplatin-sensitive and resistant syngeneic bladder cancer cell lines was validated. These findings suggest that BCOs can be used as a sensitivity screening method for drugs that could be beneficial for individualized treatments for patients.

Grassi et al. [33] studied the effects of various targeted drugs on ccRCC organoids such as SU11274, foretinib, cabozantinib and the combination of levavantinib and everolimus. ccRCC organoids were sensitive to SU11274 and foretinib and had reduced pAKT and pERK gene expressions. These two genes are important in regulating cell proliferation and arrest. However, only foretinib could sustainably induce cleaved-Caspase 3 activation, suggesting that it could sustainably induce apoptosis in ccRCC organoids. Therefore, organoids can be used as a new model to study targeted therapeutic drugs and their mechanisms.

Furthermore, organoids can be a useful model for assessing drug cytotoxicity. Cisplatin, a widely used cancer chemotherapy drug, is speculated to be nephrotoxic. It induces apoptosis in primary tubules and impairs the migratory capacity of endothelial cells. Grassi et al. [33] exposed kidney organoids to different doses of cisplatin for 48 and 72 h. Using high-resolution imaging, Western blotting and immunofluorescence, it was found that 20 and 100 μM cisplatin did not affect the structure of organoids after 48 h of treatment, whereas a concentration of 100 μM exhibited cleaved-Caspase 3 activations and apoptosis after 72 h. Moreover, only one kidney cell subtype, which co-expressed with LTL and WT1 antigens, was sensitive to cisplatin, and only kidney tubular cells were damaged.

### TME

TME refers to the cellular environment in which cancer stem cells exist. Cancer stem cells are cancer cells that can self-renew and drive carcinogenesis[76]. The TME includes immune cells, blood vessels, extracellular matrix, cancer-associated fibroblasts (CAFs), bone marrow-derived inflammatory cells and signalling molecules [77, 78]. Interactions between malignant and non-malignant cells give rise to the TME, which influences the development of cancer [79, 80]. Non-malignant cells in the TME typically exert pro-cancer functions at all stages of carcinogenesis by stimulating uncontrolled cell proliferation [81, 82]. Contrarily, malignant cells invade healthy tissue and spread to other parts of the body through the lymphatic or circulatory system. t TME affects all aspects of cancer occurrence and development; therefore, a model that can simulate TME function and interaction is the need of the hour. Organoids are one of the most promising solutions.

CAFs are an essential component of the TME, facilitating the migration of cancer cells from the primary cancer site into the bloodstream for systemic metastasis and exhibiting paracrine and physical effects on cancer. Åkerfelt et al. [83] developed a real-time live-cell measurement platform consisting of microtissues from prostate cancer cells and CAFs in the extracellular matrix. Using this platform, CAFs were observed to promote cancer growth and invasion in the organoids. Interestingly, the focal adhesion kinase inhibitors Y11 and PF-573228 selectively disrupted cancer-CAF interactions and inhibited cancer growth and invasion without significant cytotoxicity. Therefore, organoids can be co-cultured with relevant cells in the extracellular matrix to study cancer-matrix interactions.

PCOs co-cultured with bone marrow stromal cells (BMSCs) are a good model to study prostate cancer metastasis and hormone therapy resistance. Dhimoleaet et al. [84] established this model and reported that IL-6 secreted by BMSCs induced the hormone-independent growth of PCOs through the activation of the JAK/STAT signalling pathway. Additionally, the model was reported to be less sensitive to enzalutamide. Interestingly, androgen sensitivity was restored in PCOs when the IL-6/JAK/STAT signalling pathway was blocked with ruxolitinib or neutralizing antibodies, indicating that IL-6 plays an important role in the TME of mCRPC.

Although current PDOs consist of immune effectors in the TME, including peripheral immune system factors essential for optimal anticancer immunity, the immune components from the lymph nodes or blood were not combined to mimic the two-way communication between cancers and peripheral immune cells. The co-culture of PDOs with endogenous, syngeneic tumor-infiltrating lymphocytes (TILs) as a cohesive unit has been particularly elusive. James et al. [85] used an air–liquid interface (ALI) method to propagate PDOs from 100 human ccRCC biopsies or syngeneic immunocompetent mice as cancer epithelia with native embedded immune cells (T, B, NK and macrophages). Therefore, the association in vivo between native TILs and cancer cells was preserved in vitro, which was validated using the MHC tetramer detection of cancer antigen-specific T cells. Furthermore, the PDOs accurately preserved the original cancer T cell receptor (TCR) profile, which was confirmed using an a10x Chromium single-cell immunoanalysis of the 5' GEX and TCR profiles of original cancer and corresponding organoids. Importantly, both human and mouse cancer organoid TILs functionally exhibit activation, expansion and cytotoxic responses to PD-1/PD-L1 checkpoint blockade, which was validated using a rapid 7 day assessment. ALI PDOs can be used to investigate the substantial benefits of expanding immunotherapy, assess patient response to immunotherapy and facilitate the development of personalized treatments. However, further studies are required to elucidate how to prolong the preservation time of TILs and other immune cells co-cultured with PDOs. A series of prospective studies using microfluidic culture methods can be conducted to determine the clear correlation between PDOs and patient immunotherapy response, thereby achieving precise and personalised cancer treatment.

### Investigation of the original cancer cells

Cancer stem cells have been reported to quantitatively correlate with cancer invasion and metastasis and have increased resistance to chemotherapy, radiotherapy and immunotherapy [86, 87]. Therefore, establishing stem cell models of cancer origin can help to identify effective prognostic biomarkers, understand the potential mechanisms of cancers and guide the treatment and management of individuals. Advances in organoid cultures provide a new experimental platform for studying the origin of urological cancers.

Chua et al. [88] constructed a mouse prostate organoid culture system using prostate stem cells expressing Nkx3.1 under castration resistance. Compared with a previously developed culture system, serum was added to the medium along with a series of other growth factors. Moreover, mouse PCOs were created using a prostate cancer luminal cell line, with the organoids expressing functional androgen receptor signalling and exhibiting drug responses similar to the original cancer. Lineage tracing showed that luminal cells facilitate the organoids formation and generate basal cells in culture, demonstrating the stem cell properties of luminal cells. In 2015, a study using a Pten-Tp53-null prostate cancer mouse model also suggested that luminal cells could be prostate cancer stem cells. The organoids derived from luminal cells form adenocarcinomas or have multilineage cancer tissue phenotypes [89]. Additionally, Liu et al. [90] reported that low CD38 expression can be used as a biomarker for identifying luminal cells. Luminal cells with low CD38 expression were four to five times more enriched in organoids than those with high CD38 expression. Furthermore, CD38-low-expressing luminal organoids expressing both luminal (CK8) and basal (CK5 and p63) markers can regenerate the prostate in vivo. Additionally, CD38-high and CD26-positive luminal cell populations exhibited low organoid-forming activity, with 99% of cells lacking stem cell characteristics. However, some scholars believe that both basal and luminal cells are stem cells of prostate cancer. Park et al. [91] overexpressed MYC and activated AKT1(myrAKT1) in human prostate basal and luminal cells followed by prostate regeneration and transformation assays. MYC-myrAKT1-transduced luminal xenografts exhibited well-differentiated acinar adenocarcinomas whereas basal xenografts were more histologically aggressive, indicating that both basal and luminal cells can respond to the same oncogenic lesions to initiate tumorigenesis but with different cancer phenotypes.

The progenitor cell of bladder cancer remains elusive. Choi et al. [92, 93] reported that the basal type was more aggressive and metastatic. However, it had a lower survival rate than luminal bladder cancer and was more sensitive to cisplatin-based chemotherapy. Additionally, bladder cancer proliferating cells showed basal type characteristics in humans, suggesting that basal cells could be the origin cells of bladder cancer. The identification of bladder cancer stem cells has been inconclusive. A study describes basal urothelial cells expressing Sonic Hedgehog (Shh) and Cytokeratin-5 (Ck5) in Shh^CreER/WT^ and R26^mTmG/WT^ mouse-derived organoids, which include pluripotent stem cells that are capable of self-renewing and regenerating all cell types within the urothelium in response to bacterial infection or chemical damage in the bladder [54]. On injury, Shh expression in these basal cells increases and consequently increases Wnt protein stromal expression, which in turn stimulates urothelial and stromal cell proliferation. Further, single Shh-expressing cells formed cyst-like organoids after 5–7 weeks of 3D culture. The structure resembled the bladder, containing multiple layers of epithelial cells with Ck5- and Shh-expressing cells in the outer layer and a luminal space that expresses neither Ck5 nor Shh in the inner layer. Moreover, single cells from these organoids are able to self-renew and generate new organoids in subsequent cultures, indicating that Shh-expressing basal urothelial cell populations include pluripotent stem cells capable of self-renewal and differentiation. The origin and formation of bladder cancer stem cells remain unknown [94]; however, 3D-based organoid culturing could provide a new model for studying this topic [95].

### Precision medicine

Intratumoral heterogeneity indicates that a single biopsy may not contain all cancer clonal lineages. CTCs may contain unsampled foci that could provide further genomic information. Gao et al. [40] successfully established cancer organoids using a small amount of CTCs obtained from the peripheral blood of patients with prostate cancer. To the best of our knowledge, this is the only successful case of organoid extraction from blood samples reported so far. However, the inability to extract sufficient CTCs from blood samples limits the development of CTC-derived organoids. In a recent study, Lisanne et al. [96] pioneered the use of diagnostic leukotomy (DLA) to increase CTCs in patients with metastatic prostate cancer. This method provides a rich source for culturing organoids and enabling liquid biopsies.

Currently, the organoids constructed are mainly derived from cell lines or cancer tissues. In the past few years, studies have reported urine cells to possess the ability of multipotent differentiation [97] and stem cell marker expressions, such as CD44 and CD29 after culturing in the media [98]. For the first time, Tatsuya et al. [99] established urine cancer stem cells derived PCOs from dogs with prostate cancer. Immunofluorescence staining was performed to identify the cell components of urine sample-derived organoids, revealing the expression of the epithelial cell marker E-cadherin in the organoids. Interestingly, the expression of fibroblast cell marker (vimentin), myofibroblast marker (α-SMA), a leukocyte marker (CD45)and proliferating cell marker (ki67) was observed in the surroundings of organoids. These results suggest that urine-derived organoids could partially recapitulate the TME of original cancer. Moreover, testing the effect of various anticancer drugs on organoids revealed that docetaxel reduced cell viability; GLI-1 inhibitor GANT61 increased the radiosensitivity of organoids.

In 2019, Mohamed et al. [100] used the same method to generate dog bladder cancer organoids using urine samples. The expression levels of MMP28, CTSE, CNN3, TFPI2, COL17A1 and AGPAT4 have been specifically upregulated in dog BCOs. In previous studies, the expression level of MMP28 was positively associated with human bladder cancer grade, and the overexpression of CTSE has been associated with non-invasive bladder cancer [101, 102]. These studies lay the foundation for the establishment of human urine-derived cancer organoids and contribute to the discovery of novel biomarkers for human urological cancers.

Sun et al. [103] used 10% kidney-specific extracellular matrix to establish human urine stem cells-derived kidney organoids. Additionally, human kidney cells (HKCs) derived organoids were established, and USCs-derived kidney organoids were demonstrated to be similar in morphology, histology and specific gene expression. Additionally, specific proximal tubule marker Aquaporin-1, kidney endocrine product erythropoietin and kidney glomerular markers Podocin and Synaptopodin were expressed in USC-organoids, demonstrating that the organoids contain a part of the kidney structure and function. GGT is expressed in proximal tubules, and it is essential to the γ-glutamyl cycle, which plays a critical role in the detoxification of xenobiotics. Its activity can be used to represent kidney cell function [104]. The GGT assay showed that the kidney function of USCs-derived organoids and HKCs-derived organoids was impaired by aspirin, penicillin G and cisplatin in a dose-dependent manner. In the context of precision medicine, this non-invasive method of constructing organoids can be used to assess drug sensitivity and tolerance and facilitate the development of personalized treatment.

### Single-cell sequencing of organoids

**Fig.4:**
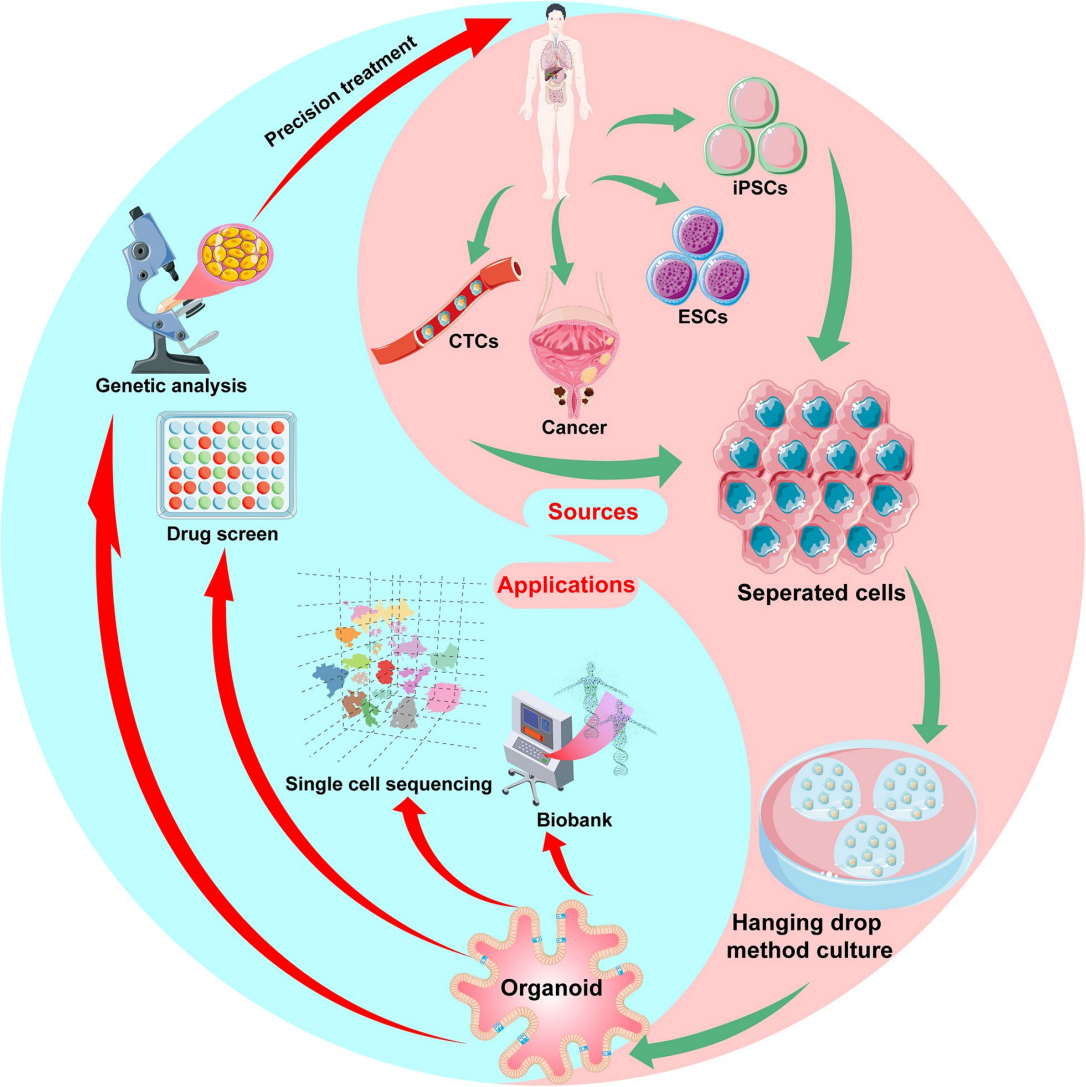
Summary of organoid establishment, sources and applications. Digest patient-derived cell lines, cancer specimens and stem cells into separated cells. Then, culture as patient-derived organoids (PDOs) by the hanging drop method. The PDOs can be used to build a biobank and perform single-cell sequencing, drug screening and genetic analyses. These results will aid in the development of personalized treatment for the patient

Traditional gene sequencing reveals the molecular information of organoids at the DNA, RNA and protein levels but it can only analyse the entire sample. Moreover, it cannot achieve single-cell resolution and cannot explain the developmental trajectory and cell communication of organoids. High-throughput single-cell sequencing technology studies the development and differentiation of cells by analysing the genetic material of each cell. For example, single-cell sequencing technology can analyse the similarity between organoids and real organs at the cellular, genetic and functional level [105]; the dynamic transcription of organoids to understand the development and differentiation of the body [106]; and the interaction between cells and cell–matrix [107]. The use of single-cell sequencing in organoids has been developed in various organs [108–110], including the urologic system [111–113]. Haojia et al. [114] compared single-cell transcriptomics of 83,130 cells from 65 kidney organoids with foetal and adult kidney cells using single-cell RNA sequencing. The results showed that organoids derived from both ESCs and iPSCs yielded a diverse range of kidney cells with varying proportions. The organoid-derived cell types were immature with 10%–20% of the cells being non-renal cells and mostly neurons. By combining pseudotemporal ordering with the lineage-specific expression of transcription factors, ligands and receptors, brain-derived neurotrophic factor (BDNF) and its receptor NTRK2 were found to be expressed in neural clusters. Thereby, the off-target cell types were reduced by 90 percent by inhibiting BDNF-NTRK2 signalling. Similar approaches can be broadly applied to organoid development to reduce unwanted cell types. In 2020, Camilla et al. [115] established the first organoid biobank for paediatric cancers, which included 54 kidney tumour organoids and matched normal kidney organoids. It comprises Wilms tumour (40), malignant rhabdoid tumours of the kidney (7), renal cell carcinomas (3), congenital mesoblastic nephroma (2), nephrogenic rest (1) and metanephric adenoma (1). Using single-cell RNA sequencing and high-resolution 3D imaging, Wilms tumour-derived organoids were found to comprise multiple distinct cell types including epithelial, stromal and blastocyst-like cells. Annika et al. [23] identified a subpopulation of self-renewing renal cancer stem cells (CSCs) that could be identified by a CXCR4 + MET + CD44 + profile. Analyses of gene and protein expressions revealed the activation of Wnt and Notch signalling in these CSCs. On using single-cell sequencing to determine the degree of heterogeneity within the CSCs population, it yielded three subpopulations, two of which exhibited a high expression of markers known to be associated with stem cells and kidney development. These two populations also showed a high expression of Wnt and Notch signalling components and their corresponding target genes. Furthermore, CSCs were used to establish a renal cancer organoid. Treatment with Wnt and Notch inhibitors was found to block the proliferation and self-renewal of CSCs in organoids, suggesting that single-cell sequencing can be used to determine the heterogeneity of a cell population and contribute to the development of personalized treatments (Fig. 4).

## Challenges of organoids

**Fig.5:**
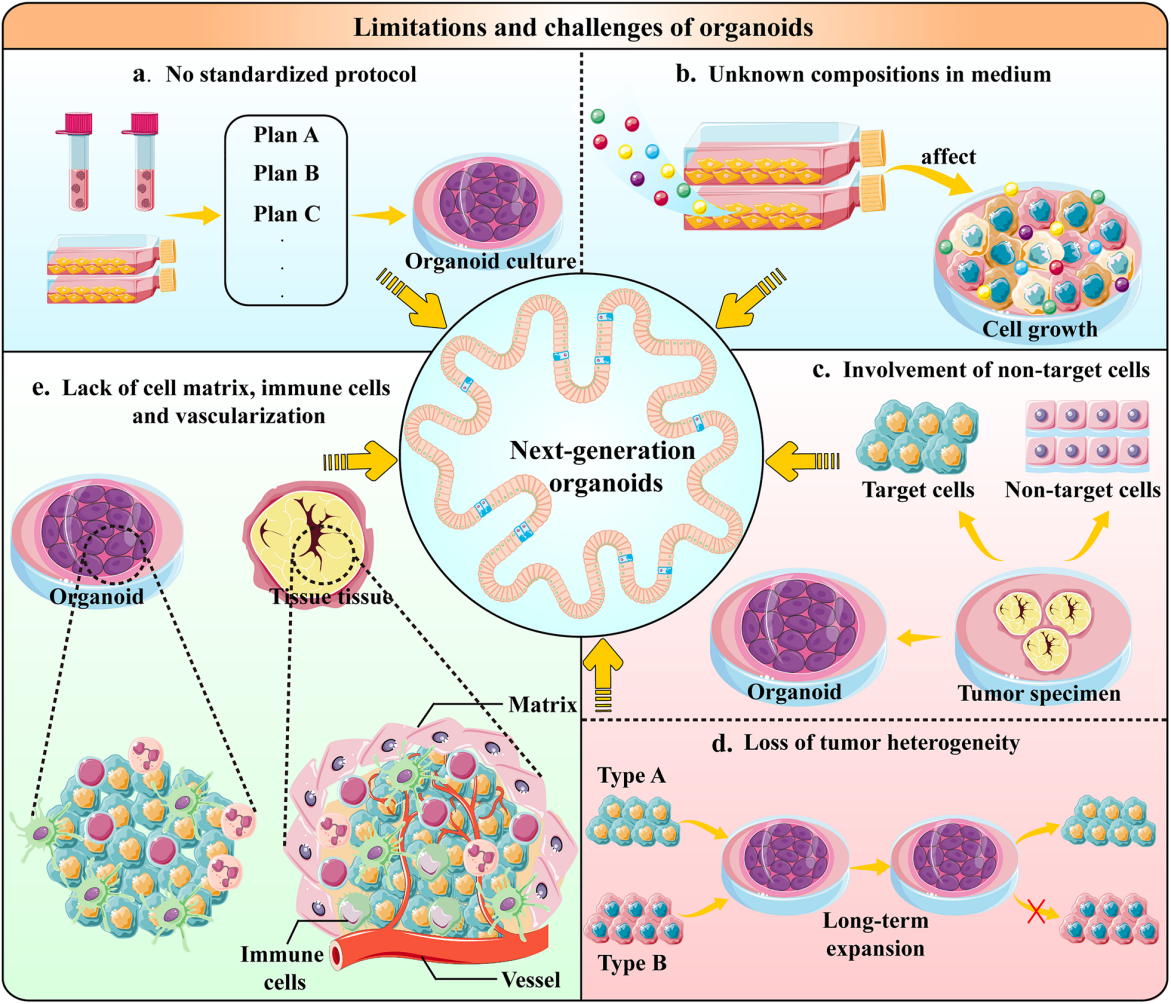
Limitations and challenges of organoids. **a**, **b** Extensive work across the scientific community is required to standardize the multiple steps involved in culturing organoids. Some exogenous additives in media may have potential effects on culturing organoids, leading to low efficiency and a short lifespan of organoid cultures. **c**, **d** The tumour specimens resected during surgery are often mixed with normal tissue cells. Therefore, the organoids established using them are likely to be contaminated with benign cells. It is yet to be determined if a loss of intertumoral heterogeneity in cancer organoids could occur upon long-term expansion. Considering these problems, organoids cannot accurately reflect the cancer characteristics, leading to inaccurate drug screening results. **e** Current organoid models still lack an intact tumour microenvironment, such as stromal cells, vascular endothelial cells and immune cells. Therefore, better co-culture systems need to be developed to reflect extracellular matrix, cell–matrix and cell-to-cell interactions

Organoids are good models for mimicking cancers and are useful for studying the reason, diagnosis and treatment of urologic cancers. However, the limitations and technical challenges of organoids cannot be ignored (Fig. 5). Organoids, as a pure epithelial culture model, lack many cellular components in the body such as stromal cells, vascular endothelial cells and immune cells [116], which are important components of the TME and have a huge impact on the biological behaviour of cancers, Additionally, these interactions affect drug sensitivity. Therefore, more co-culture systems need to be developed that incorporate multiple cell types into organoid cultures to reflect cell–matrix and cell-to-cell interactions [117, 118]. Currently, there are three main co-culture models: submerged Matrigel culture, ALI culture and microfluidic culture [119]. Organ-on-a-chip is a multi-channel microfluidic cell culture device that includes multiple cell types to model the structure and function of parental cancer [8]. By integrating organoids with organ-on-a-chip engineering, physiologically relevant microenvironments can be generated, resulting in a model that truly represents the complex characteristics of cancer progression [120]. Liu et al. [121] co-cultured human bladder cancer cells, fibroblasts, macrophages and human umbilical vein endothelial cells on a chip and screened out different chemotherapy regimens, highlighting the differences in the sensitivity of bladder cancer cells. Conventional organoid cultures also do not have a vasculature, relying entirely on their culture medium for nutrients and can therefore only grow to a certain size. However, organoid‐on‐a‐chip can build a quiescent perfused 3D microvascular network to overcome the obstacle of nutrient supply [122]. For instance, Shirure et al. [123] mixed cancer cells, lung fibroblasts and endothelial colony-forming cell endothelial cells in fibrin and seeded them in a single chamber of a microfluidic device under physiological interstitial flow conditions, ultimately generating vascularized micro-tumours. Moreover, most of the existing organoids are cultured independently with culture conditions varying for different organoids. Therefore, organoids fail to model interactions between different tissues or organs, which play an essential role in organ development and homeostasis. Recently, Novakovic et al. [124] made a breakthrough by designing a tissue-chip system in which matured human heart, liver, bone and skin tissue niches were linked by recirculating vascular flow to allow for the recapitulation of interdependent organ functions. This model can better reflect the pharmacokinetics and pharmacodynamics of drugs in vivo while enabling the exchange of cytokines, circulating cells and exosomes between different organs. This study inspired the construction of patient-personalized multi-organoid chips to explore the underlying mechanisms of cancer metastasis and the connection between multiple cancers. Furthermore, tissue engineering and the intrinsic self-organization properties of cells can be used to simulate the real structure and physiological function of organs. Nikolaev et al. [123] used scaffolds in an intestine shape to generate a ‘mini-gut’ with typical crypt structures and villi-like domains. This microdevice guides self-organizing intestinal stem cells into functional organoid-on-a-chip. Notably, a more advanced model based on organoids has been developed—assembloids–which have a multi-layered structure that can be reconstructed three-dimensionally by combining stem cells with various cell types in the tissue matrix. Kim et al. [125] established bladder cancer assembloids using 3D reconstructions of patient-derived BCO, patient-matched endothelial cells and cancer-associated fibroblasts. The reconstituted endothelial cells often developed into vascular networks that promote cancer growth, with sequencing analyses suggesting that genetic alterations in parental cancer are reflected in the assembloids. Moreover, a regulatory mechanism, the FOXA1-BMP-hedgehog axis, that affects the bidirectional differentiation of basal cells and luminal cells of the bladder cancer assembloids was elucidated, indicating a potential mechanism for generating different cancer subtypes.

Although an organoid is more convenient than a xenograft, its success rate and efficiency are lacking and vary greatly among different cancers. For example, organoid efficiencies of only 15%–20% from metastatic prostate cancer biopsy samples could be related to the overgrowth of cancer-associated spindle cells. The efficiency of producing BCOs is about 30–70% [15, 126]. Organoids from ccRCC are less efficient than bladder cancer, which could be attributed to the severe necrosis that affects renal cancer tissue [52]. The low success rate of organoid cultures and the large variability among different cancer types could be due to the absence of standard procedures. Microfluidic organoid-on-a-chip technology typically uses polymeric materials, such as polydimethylsiloxane, allowing for easily controlling spatiotemporal flow. Additionally, nutrient supply, geometry and shear stress can be easily controlled in the organoid-on-a-chip platform, which enables the standardization of a culture protocol.

Currently, there are many types of media for organoids. Some exogenous additives may have potential effects on cancers, requiring the continuous optimization of the media. Matrigel is an essential substance in current media but its composition is undefined. It has limited the utility of organoids for drug development and regenerative medicine due to its mouse tumor-derived origin, unclear composition, batch-to-batch variation and high cost. Several studies have developed other scaffold-based technologies or designed novel chemical materials as potential alternatives to the widely used Matrigel. For example, Kim et al. [127] demonstrated that gastrointestinal tissue-derived extracellular matrix hydrogels were effective substitutes for Matrigel in gastrointestinal organoid culture. Gastrointestinal extracellular matrix hydrogels enabled long-term subculturing and transplantation of organoids by providing gastrointestinal tissue-mimetic microenvironments. Furthermore, Prince et al. [128] reported a nanofibrillar hydrogel (EKGel) with controllable stiffness, resulting from a reaction between chemically modified cellulose nanocrystals and gelatin. Breast cancer organoids grown in EKGel have histopathologic features, gene expressions and drug responses that are similar to those of their parental cancers. These novel materials reduce Matrigel batch-to-batch variability and contamination of mouse cells, thereby aiding in the optimization of an organoid culture.

The cancer specimens resected during surgery are often mixed with normal tissue cells. The organoids established using these specimens are likely to be contaminated by benign cells, which can affect the gene analysis and drug screening of cancer organoids. Metastatic prostate cancer organoids have been established, but establishing organoids derived from primary prostate cancer was technically challenging owing to the overgrowth of non-malignant prostate epithelial cells that were present within each sample [40]. Currently, there is no good method to obtain pure tumour organoids. Existing methods for selecting cancer cells are based on the most common mutations and growth phenotypes or establishing organoids from single cells, which all have significant limitations [129–132]. Thus, obtaining pure tumour organoids remains a big challenge.

It is yet to be determined if a loss of intratumoral heterogeneity in cancer organoids could occur upon long-term expansion. To enable a stable and spontaneous generation of differentiated cells, unconventional organoid culture methods, such as using the ALI or creating artificial growth factor gradients, are needed [133, 134]. Additionally, Qu et al. [135] found that intestinal organoids express injury regeneration genes and grow more complex budding structures when using an eight-component culture system. These features are very similar to the hyperplastic crypts following injury, hence, the name ‘Hyper-organoid’. Compared with traditional organoids, the expansion capacity of Hyper-organoids has been greatly enhanced. After five generations of continuous culturing, the number of intestinal stem cells increased by 10,000 times. Furthermore, Hyper-organoids are capable of long-term expansion and maintain genomic stability.

## Conclusions and prospects

Cancer organoids can effectively retain the molecular, cellular and histological phenotypes of original cancer and maximally maintain patient-specific cancer heterogeneity compared to cell lines and PDXs. With the increasing establishment of biobanks and the diversification of sources, cancer organoids, as a preclinical pre-model, are widely used for drug screening, biomarker research and personalized treatment development. Although cancer organoids face many challenges, microfluidic-based organoid-on-a-chip enables the precise regulation of TME and the standardization of culture procedures, allowing for the development of more comprehensive cancer model systems. Complex cancer organoid systems, including immune cells, vascular networks and other cytokines, may be developed in the future, and the molecular characterization of cancer phenotypes can be further investigated using this technology. Moreover, an integrated system containing various urological cancer organoids chips is expected to be established, allowing for a comprehensive understanding of cancer metastasis and interaction between cancers. Overall, the combination of organoids and multiple technologies is speculated to revolutionize the traditional model of cancer research, thereby advancing the development of precision therapy.

## Data Availability

Not applicable.

## Abbreviations

2D: Two-dimensional
TME: Tumor microenvironment
PDXs: Patient-derived xenografts
3D: Three-dimensional
PDOs: Patient-Derived Organoids
Lgr5: G protein-coupled receptor 5
EGF: Epidermal growth factor
BMP: Bone morphogenetic protein
Rock: Rho-related protein kinase
FGFs: Fibroblast growth factors
ccRCC: Clear cell renal cell carcinoma
CTCs: Circulating tumor cells
PCOs: Prostate cancer organoids
NEPC: Neuroendocrine prostate cancer
RP: Radical prostatectomy
TURP: Transurethral resection of prostate
DHT: Dihydrotestosterone
PGE2: Prostaglandin E2
FBS: Fetal bovine serum
BCOs: Bladder cancer organoids
HER3: Human epidermal growth factor receptor 3
ESCs: Embryonic stem cells
iPSCs: Induced pluripotent stem cells
hiPSCs: Human induced pluripotent stem cells
RT-PCR: Reverse transcription-polymerase chain reaction
CRPC: Castration-resistant prostate cancer
ADT: Androgen deprivation therapy
mCRPC: Metastatic castration-resistant prostate cancer
DDR: DNA damage response
EMT: Epithelial-mesenchymal transition
PARP: Poly ADP ribose polymerase
ECM: Extracellular matrix
CAFs: Cancer-associated fibroblasts
BMSCs: Bone marrow stromal cells
TILs: Tumor-infiltrating lymphocytes
ALI: Air–liquid interface
TCR: T cell receptor
myrAKT1: Activated AKT1
Shh: Sonic Hedgehog
Ck5: Cytokeratin-5
DLA: Diagnostic leukotomy
kECM: Kidney-specific extracellular matrix
USCs: Urine stem cells
HKCs: Human kidney cells
AQP1: Aquaporin-1
EPO: Endocrine product erythropoietin
BDNF: Brain-derived neurotrophic factor
CSCs: Cancer stem cells
RCO: Renal cell carcinoma organoid
